# The Potential Effect of Sugar-Sweetened Beverages Tax on Obesity Prevalence in Tanzania

**DOI:** 10.24248/eahrj.v7i2.743

**Published:** 2023-11-30

**Authors:** Martin Julius Chegere, Tunguhole Jires, Songora Fortunata, Masalu Emmanuel, Ngoma Twalib, Mayige Mary, Lasway Jovin

**Affiliations:** a University of Dar es Salaam, Dar es Salaam, Tanzania; b Sokoine University of Agriculture; c Economic and Social Research Foundation; d Institute of Tax Administration; e Muhimbili University of Health and Allied Sciences; f National Institute of Medical Research

## Abstract

**Background:**

Obesity and the associated non-communicable diseases contribute significantly to the disease burden in Tanzania. Obesity can be attributed to the consumption of Sugar Sweetened Beverages (SSB) due to their high sugar content that leads to high caloric intakes. This study estimates the effect of SSB tax on the prevalence of obesity.

**Methods:**

A mathematical model that compares the reference population which is unchanged and a counterfactual population in which tax intervention has been introduced is developed. Changes in price and consumption of SSBs, and subsequent changes in energy intake are applied to estimate the body mass change by age groups. The change in body mass by age groups is merged with the reference population to estimate changes in body mass index and obesity.

**Results:**

Imposing a 20% SSB tax in Tanzania is estimated to reduce the average overall energy intake by 76.1 kJ per person per day. This change is associated with an overall reduction of prevalence of obesity by 6.6%; and by 12.9% and 5.2% in adult males and adult females, respectively. The number of obese people will potentially decrease by about 47,000 among adult males and about 85,000 among adult females from the current levels.

**Conclusions:**

The SSB tax is a potential strategy to complement efforts to reduce obesity prevalence in Tanzania. The revenue generated from the tax should be channelled towards public health promotion programs.

## BACKGROUND

Obesity is a growing global challenge in terms of prevalence, health outcomes and economic burden. The World Health Organisation (WHO) estimated that 39% (1.9 billion) of adults aged 18 years and above were overweight and about 13% (650 million) of the world's adult population (11% of men and 15% of women) were obese.^1^ The WHO report also shows that an estimated 41 million children under the age of 5 years were overweight or obese. Once considered a high-income country problem, overweight and obesity are now on the rise in low- and middle-income countries, particularly in urban settings. In Africa, the number of overweight children under 5 has increased by nearly 50 per cent in 2014 since 2000.^2^

The trend of obesity and overweight prevalence in Sub-Saharan Africa has continued to increase among women and people dwelling in urban populations.^3,4^ In Tanzania, the trend of obesity prevalence rate has increased drastically, for both men and women, from 5.9% in 2014 to 8.4% in 2016.^5^ Being overweight and obese contributes to high prevalence rate of people with Non-Communicable Disease (NCD) risk factors such as diabetes, cardiovascular diseases (CVDs) and cancer, and the overall health effects.^6^

Obesity and the associated NCDs contribute significantly to the disease burden especially among the adult populations globally, including Tanzania.^7–9^ It is estimated that in Tanzania, the trend of deaths due to NCDs, has increased from 19.5% of all deaths in 2000 to 25.8% in 2010 and further increased to about 32.9% in 2016.^1^ According to the Global Burden of disease report, ^10^ metabolic risks contributed 17.3% of total deaths (both sexes, all ages), of these 4.95% are directly attributed to high body mass index. Metabolic risks contributed to 7.36% of total disability-adjusted life years (DALYs) in 2019, of these 2.55% of total DALYs were directly attributed to high body mass index.

The consumption of Sugar Sweetened Beverages (SSBs) which have high sugar content that leads to high caloric intake is strongly linked to the increase in obesity. ^11–13^ In addition to this, increasing SSBs consumption leads to other NCDs such as CVD, type II diabetes, dental caries and metabolic syndrome.^14^ Furthermore, through habitual consumption of higher caloric intake from SSBs in childhood the risk of obesity can persist in adulthood.^15^ The catastrophic expenses on cost of care, loss of income and other indirect costs for treating NCDs puts much financial burden on families. As more people suffer and die from costly chronic NCDs and fall into poverty, consequently, the government is expected to shoulder the tremendous cost of treating NCDs.

A number of studies have analysed the effect of SSB tax on consumption of SSBs and found that the tax reduces consumption of SSBs.^16–18^ Several studies have used mathematical simulation models to analyse the impact of SSBs tax on SSBs consumption, subsequent caloric intake, and obesity prevalence.^16,18^ Countries, such as South Africa, United Kingdom (UK), Ireland, India, Brazil, Denmark, Hungary, France etc., have used fiscal policy measures such as taxes on SSBs as policy intervention to reduce obesity as a chronic risk factor for NCDs through reduction of SSBs consumption.^16,19–23^ However very few studies have been conducted in developing countries where consumption patterns, and tax structure and mechanism are different from those in developed countries.

Over the years, Tanzania has been increasing tax on alcohol and SSBs with the aim of generating revenues. Little were those fiscal measures implemented as corrective tax with the aim to discourage consumption of alcohol and SSBs. Evidence on how taxation of SSBs would reduce SSBs consumption and consequent reduction in obesity prevalence in Tanzania remains unkown. This study, therefore, seeks to fill that gap by investigating the potential impact of SSBs tax on obesity prevalence in Tanzania using mathematical simulation models.

## METHODS

**FIGURE 1:**
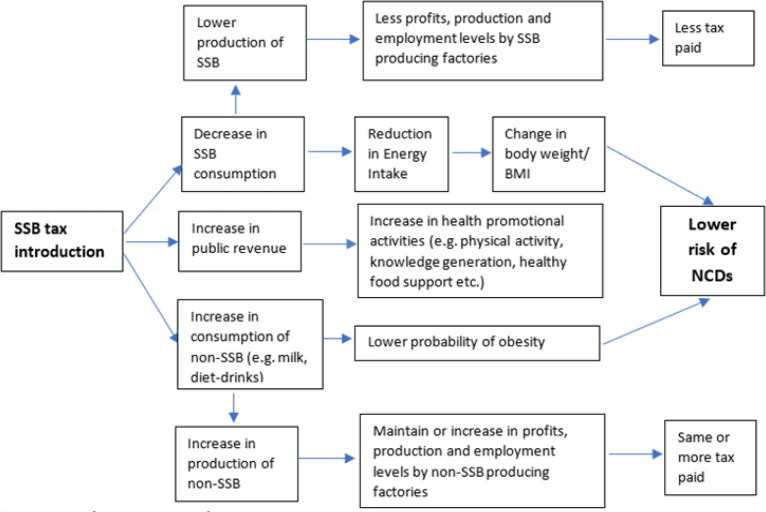
The Logical Pathway of the Impact of SSB Tax on Obesity

Source: Authors' Construction

The imposition of SSBs tax is expected to be passed to consumers through higher prices of SSB products. Assuming that SSBs are normal goods, the income and substitution effect of price increase will lead to lower consumption of SSBs according to the price elasticity of demand of the SSB product. Changes in the amount of SSB consumed will lead to changes in total intake of calories which in turn will lead to change in energy resulting in changes in body weight and eventually the change in Body Mass Index (BMI), as a measure of obesity and overweight (Figure 1). Obesity and overweight follow the standard BMI classification as: Underweight (BMI < 18.5); Normal weight (BMI 18.5 – 24.9); Overweight (BMI 25.0 – 29.9) and Obesity (BMI 30+)

A mathematical simulation model is constructed and executed using Microsoft Excel and STATA software, to estimate the effect of SSB tax on obesity in Tanzania. The effects of different tax rates on the prevalence of obesity are tested. The analysis presents a partial equilibrium effect and is disaggregated according to gender and across age groups to explore heterogeneities.

### Data and Assumptions

#### Pass Through Rate

Pass through rate is the proportion of tax change that is passed on to buyers in form of price changes. The SSB tax once introduced may be passed on in full to consumers, or manufacturers and retailers may absorb some of the tax by reducing price margins. In some other cases a pass-through rate may even exceed 100%. Various research informed the pass-through rate to be assumed. The study by Besley and Rosen using data from the USA suggested that the pass through-rate was in excess of 100% for soft drinks.^24^ The study by Berardi, Sevestre, Tepaut and Vigneron^25^ showed that a ‘soda tax’ was fully shifted to soda prices and almost fully shifted to the prices of fruit drinks. However, the study of the Irish tax on SSBs in the 1980s^26^ suggests a pass-through rate of less than 100%. In cases where there is uncertainty with the pass-on rate, it is considered reasonable to assume a pass through rate of 100%.^27^ The study by Briggs and others co-authors^20^ assessing the impact of a 10% SSB tax on obesity assumed a pass through rate of between 80% and 100% whereas the study by Manyema and other co-authors in South Africa^16^ assumes a pass through-rate of 100%. Since we do not have data for Tanzania it seems reasonable to assume a pass through rate of 100%.

#### Price Elasticities

Price elasticity refers to the rate of response of the quantity of a good demanded when the price increases. Own-price elasticity measures the change in demand that occurs for a good in response to price changes of the same good. Cross-price elasticity is the change in purchases that occur for a good in response to price changes of another good. Price elasticity estimates from the Economic and Social Research Foundation (ESRF) survey data are used. The survey collected data on how much SSB and their substitute's individuals consumed in the past seven days, and then asked how much they would consume in case the price rises by 20%. The responses were then used to calculate the elasticities. The reference period of one week was used to reduce information and recall bias.

The ESRF survey data collected information from the following groups: households, patients, caretakers, and health workers. The field work was conducted in eight (8) regions, namely: Dar es Salaam, Dodoma, Arusha, Mbeya, Tanga, Mwanza, Mtwara, and Kigoma representing each of the geographical zones in Tanzania. In each region, one district was randomly selected from which a random selection of households and patients was then done. For health workers, the sampling was purposive to get one who could provide the best required information. Different questionnaires and interview guides were developed for each category of respondents depending on the type of information sought from each group. The number and distribution of targeted samples that have been collected by regions can be seen in Table 1.

**TABLE 1:** Selected Regions with Sample Size From the Fieldwork

Region	Local Government Authority	Household (Survey)	Caregivers (Survey)	Patients (Survey)	Health workers (Key Informant)
Arusha	Karatu District Council (DC)	50	14	7	1
Tanga	Tanga City Council (CC)	50	14	7	1
Mwanza	Ilemela DC	50	14	7	1
Kigoma	Kasulu Town Council (TC)	50	14	7	1
Mtwara	Masasi DC	50	14	7	1
Mbeya	Mbeya CC	50	14	7	1
Dodoma	Dodoma Municipal Council (MC)	50	14	7	1
Dar es Salaam	Ilala DC	50	14	7	1
TOTAL RESPONDENTS		450	112	56	8

Source: ESRF Survey, 2019

#### Prevalence of Obesity in Tanzania

Obesity was measured by BMI. BMI was estimated from the anonymized dataset of the third wave of the Tanzania National Panel Survey (TNPS) which was conducted in 2012/13. These TNPS were implemented by Tanzania's National Bureau of Statistics (NBS) and are part of the Living Standard Measurement Studies initiated and partially funded by the World Bank. The survey data was collected from October 2012 to November 2013. The TNPS is a national level longitudinal survey designed to provide data from the same households over time in an attempt to understand poverty dynamics and to evaluate policy impacts in the country. The TNPS is based on a stratified, multi-stage cluster sample design. The sampling frame for the third wave is the 2002 Population and Housing Census, more specifically, the National Master Sample Frame, which is a list of all populated enumeration areas in the country. The dataset contains information for 25,412 individuals from 5,050 households. Among the individuals only those who were 15 years of age or above were considered for the analysis (from the sample, 13,239 individuals were 15 years or above). The TNPS household survey aimed to collect household and individual data as well as anthropometric measures. Data was cleaned and coded using STATA Version 14. For analysis, the sample was disaggregated by age and sex. BMI for each adult whose measurement was taken was computed as weight in kilograms divided by the square of height in metres. Extreme BMI values falling below 10 and above 60 were excluded from the sample used for the analysis.

### Modelling

#### Step 1 – Effect of SSB tax introduction on SSB consumption.

Valoric tax rate of 20% and 100% pass through rate are used to estimate a price rise, which together with own-price elasticity for SSBs are used to estimate the percentage change in purchasing and hence consumption of SSBs. The own price elasticities for SSBs and the cross elasticities for SSB substitutes are used to estimate the changes in their consumption. Consumption of beverages was measured in milliliters per person per day.

#### Step 2 – Effect of change in SSB consumption on energy intake.

Average calorie density estimates for each drink are used to convert change in volume consumed to change in energy intake, assuming the percentage change in energy intake to be the same as percentage change in volumes of SSB and their substitutes consumed. The changes in caloric intake for each beverage type are assumed to give the net change in energy intake. The different baseline beverage consumption levels by age and sex combined with the percentage change in consumption give different absolute estimates for change in amount consumed by age and sex.

#### Step 3 – Effect of change in energy intake on body mass index and obesity prevalence.

Change in body mass is estimated using mathematical relationships which have been established by previous studies. It is assumed that a new ‘steady state’ body mass is achieved if either total energy intake and/or level of physical activity change.^21^ In the modelling conducted in this study, we assume that the average level of physical activity is unchanged, so all the derived changes in body mass come from change in energy intake. The study adopts the conversion rate used by Manyema and co-authors^16^ which requires a daily increase in energy intake of 94 kJ/day to change a body mass of adults in equilibrium for 1 kg.^28^ On average, half the body mass change can occur in one year and 95% of the change in three years.^29^ This change in average body mass is converted to change in average BMI in a particular age group by using the height of individuals in the age group using the third wave of TNPS data.

Apart from SSB consumption, there are other factors which may also correlate with obesity. The main confounders are physical activity and diet, others include age, sex, socioeconomic status, location (rural urban). While the data used cannot account for all confounding factors, we account for some by disaggregating the analysis by age groups and sex.

## RESULTS

### Baseline Consumption

Baseline consumption data from ESRF survey show that on average adults in the sample consume 150.8 ml of SSBs, 3.4 ml of diet drinks, 123.6 ml of milk and 196.4 ml of tea or coffee a day. Adults in age group 25–34 years consume more SSBs (213 ml per day) compared to any other group and then consumption declines with age, while those who are 65 years and above consume the least amount of SSBs (on average, 98.5 ml per day) (Table 2).

**TABLE 2:** Baseline Consumption of SSBs

Age	Mean	SSB Confidence interval 95%	Mean	Milk Confidence interval 95%	Diet drinks Mean	Confidence interval 95%	Tea or Coffee Mean	Confidence interval 95%
15–24	135.7	[73.5, 197.8]	75.7	[14.2, 137.1]	0.0	0	204.4	[143.4, 265.5]
25–34	213.5	[124.8, 302.3]	148.0	[91.1, 204.9]	1.0	[−1.0, 3.2]	176.2	[135.9, 216.5]
35–44	158.4	[108, 208.8]	94.6	[53, 136.1]	1.0	[−1.0, 3.1]	173.4	[134.6, 212.1]
45–54	130.3	[86.3, 174.3]	105.8	[53.9, 157.7]	6.1	[−0.4, 12.8]	236.0	[184.7, 287.4]
55–64	123.8	[47.5, 200.2]	181.5	[97.3, 265.6]	5.3	[−2.4, 13.0]	210.3	[136.6, 284.1]
65+	98.5	[56.1–140.9]	145.9	[57.2, 234.5]	8.3	[−8.4, 25.1]	186.2	[127.7, 244.6]

Source: ESRF Survey, 2019

The baseline consumption of SSBs substitutes is on average 323.4 ml per day. A great part of this consumption is tea or coffee (196.4 ml). Those above 45 years consume relatively more of SSB substitutes compared to those below.

### Change in Daily Energy Intake and Body Mass

We assume a pass through rate of 100% which implies that if a 20% valoric tax is imposed on SSBs, price will also increase by the same percent (20%). The change in price of SSBs will translate into change in consumption of SSB. The magnitude of this change will depend on the price elasticity of the particular product. The change in price of SSBs may also affect the consumption of SSB substitutes; the magnitude of which will depend on the cross-price elasticity.

Table 3 presents the own- and cross-price elasticity computed from ESRF survey. The own-price elasticity of SSB products is negative implying that imposing a tax on SSB decreases the amount of SSB purchased and consumed. This is because the price of SSBs becomes relatively higher compared to their substitutes (substitution effect), and/or because given the income, the purchasing power decreases because of higher level general price (Income effect). With the exception of diet drinks, the cross elasticity of SSB substitutes is positive, implying that the increase in price of SSBs will increase consumption of SSB substitutes.

**TABLE 3:** The Own- and Cross-price Elasticities of SSB and Their Substitutes

Beverage	Mean own-price elasticity	Mean cross-price elasticity	95% Confidence intervals
Soit drinks	−2.4		[−2.78, −2.02]
Energy drinks	−2.7		[−3.53, −1.96]
Fruit juice	−1.9		[−3.46, −0.31]
Milk		0.8	[0.4, 1.22]
Diet drinks		−0.3	[−1.71, 1.08]
Tea/Coffee		2.4	[1.57, 3.22]

Source: ESRF Survey, 2019

Table 4 presents the impact of SSB valoric tax of 20% on total daily energy intake. The average overall change in energy intake is 76.14 KJ per day per person. The changes in energy intake are statistically significant for all age groups for the overall sample and among males but significant only for some age groups (25–34 years and 55–64 years) among females. Changes in energy intake are greater among males relative to females. The reduction in energy intake shows variation by age but without a clear and consistent pattern, reflecting the consumption pattern. The reduction in energy intake is higher among males in age group 25–34 years (288.6 KJ per day) compared to any other group, and is the lowest among females of age 35–44 years (159 KJ per day) (Table 4).

**TABLE 4:** Estimated Changes in Energy Intake

Change in mean energy intake in Kj/person/day [95% confidence interval]						
Age group		Male		Female		Overall
15–24	−59.74	[−116.59, −2.88]	−77.91	[−169.45, 13.63]	−72.20	[−135.51, −8.89]
25–34	−288.59	[−546.91, −30.28]	−74.47	[−113.65, −35.30]	−111.94	[−166.70, −57.19]
35–44	−83.02	[−157.58, −8.47]	−40.99	[−100.86, 18.87]	−57.54	[−103.53, −11.54]
45–54	−88.63	[−144.69, −32.57]	−49.68	[−111.27, 11.92]	−66.99	[−108.65, −25.32]
55–64	−105.34	[−197.57, −13.10]	−61.74	[−106.35, −17.13]	−77.98	[−120.76, −35.21]
65+	−70.77	[−130.31, −11.22]	−65.06	[−133.04, 2.93]	−68.32	[−111.17, −25.46]

Source: ESRF Survey, 2019

Reductions in daily energy intake translate into reduction in body mass according to the established conversion rates. The changes in body mass presented in Table 5 are directly proportional to change in energy intake. So, similar to the changes in energy intake, the changes in body mass are statistically significant for all age groups for the overall sample and among males; but significant for females of age groups 25–34 years and 55–64 years only.

**TABLE 5:** Estimated Changes in Energy Body Mass

Change in mean body mass in Kg/person/day [95% confidence interval]						
Age group		Male		Female		Overall
15–24	−0.64	[−1.24, −0.03]	−0.83	[−1.80, 0.14]	−0.77	[−1.44, −0.09]
25–34	−3.07	[−5.82, −0.32]	−0.79	[−1.21, −0.38]	−1.19	[−1.77, −0.61]
35–44	−0.88	[−1.68, −0.09]	−0.44	[−1.07, 0.20]	−0.61	[−1.10, −0.12]
45–54	−0.94	[−1.54, −0.35]	−0.53	[−1.18, 0.13]	−0.71	[−1.16, −0.27]
55–64	−1.12	[−2.10, −0.14]	−0.66	[−1.13, −0.18]	−0.83	[−1.28, −0.37]
65+	−0.75	[−1.39, −0.12]	−0.69	[−1.42, 0.03]	−0.73	[−1.18, −0.27]

Source: ESRF Survey, 2019

### Change in BMI

**FIGURE 2:**
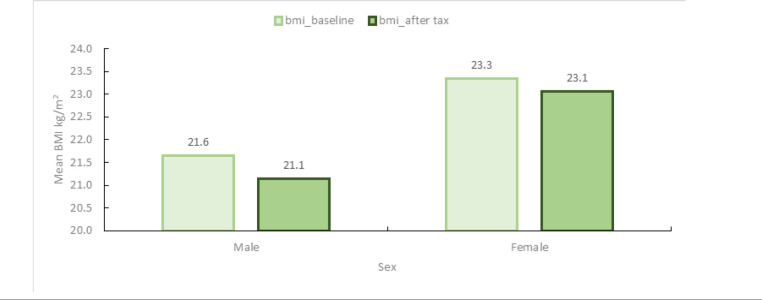
Mean BMI by Sex at Baseline and After SSB Tax

Source: Tanzania National Panel Survey (2012/13)

Figure 2 shows the mean BMI levels at the baseline and after the SSB tax intervention for both men and women based on anthropometric measures of adults above 15 years from the third wave of the TNPS.

**FIGURE 3:**
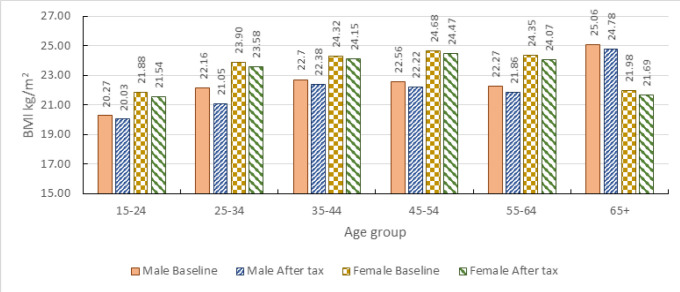
Mean BMI by Sex and Age groups

Source: Tanzania National Panel Survey (2012/13)

The baseline mean BMI is higher for females compared to males. On average, at the baseline, the BMI for males is 21.6 kg/m^2^ and for females 23.3 kg/m^2^. SSB tax of 20% leads to the decline in BMI for both men, by 0.5 kg/m^2^ (equivalent to 2.3% decrease) and women, by 0.29 kg/m^2^ (equivalent to 1.3%); and still remains higher for females after the tax (Figure 3).

Figure 3 indicates that the mean BMI levels before and after the intervention by sex and age groups.

The baseline mean BMI is higher among females compared to males in all age groups except for those who are of age 65 and above. Those in the lower age group (15–24 years) have the lowest average BMI for both males and females. Among females, the middle aged (45–54 years) have the highest average BMI; while among males the older (65+ years) have, by far, the highest average BMI compared to other age groups.

The imposition of 20% SSB tax leads to significant BMI declines for all adults in all sex and age groups (Table 6). The decline is higher among those in age group 18–29 years.

**TABLE 6:** Estimated Changes in BMI

Change in mean BMI in Kg/m2 [95% confidence interval]						
Age group		Male		Female		Overall
15–24	−0.24	[−0.241, −0.238]	−0.35	[−0.347, −0.344]	−0.30	[−0.302, −0.298]
25–34	−1.11	[−1.112, −1.101]	−0.33	[−0.327, −0.324]	−0.67	[−0.685, −0.655]
35–44	−0.32	[−0.319, −0.316]	−0.18	[−0.180, −0.178]	−0.24	[−0.241, −0.235]
45–54	−0.34	[−0.345, −0.340]	−0.22	[−0.221, −0.218]	−0.28	[−0.279, −0.272]
55–64	−0.41	[−0.415, −0.408]	−0.28	[−0.279, −0.274]	−0.34	[−0.342, −0.332]
65+	−0.27	[−0.285, −0.265]	−0.29	[−0.300, −0.286]	−0.29	[−0.292, −0.280]

Source: Tanzania National Panel Survey (2012/13) and author's calculations

### Effect on Obesity

**FIGURE 4:**
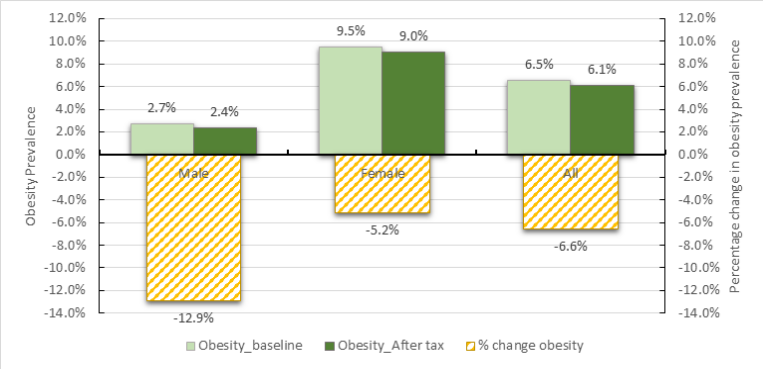
Obesity Prevalence Before and After SSB tax, Overall and by Sex

Source: Tanzania National Panel Survey (2012/13) and author's calculations

The prevalence of obesity during the baseline is 6.5% and it is more pronounced among females where 9.5% are obese compared to males who are at 2.7% as shown in Figure 4. The mathematical model projects the overall prevalence of obesity to go down by 0.4 percentage points which is equivalent to 6.6% change. Obesity declines more among males, going down by 0.3 percentage points, equivalent to 12.9% change. Obesity among females declines by 0.5 percentage points which is equivalent to 5.2% change.

**FIGURE 5:**
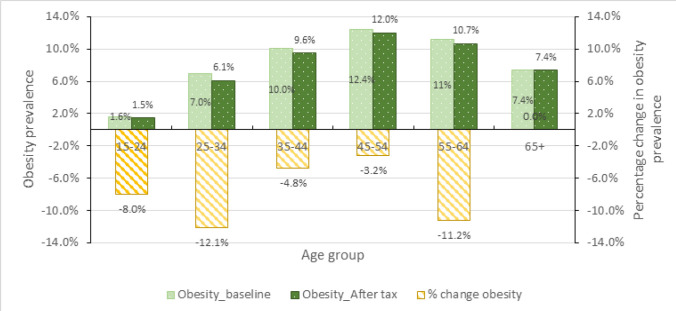
Obesity Prevalence Before and After SSB tax, by Age Groups

Source: Tanzania National Panel Survey (2012/13) and author's calculations

**FIGURE 6:**
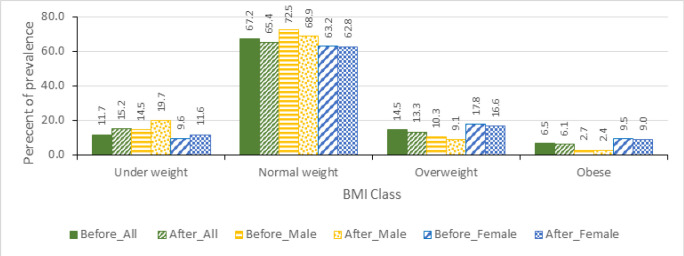
BMI Classification Groups Before and After SSB tax From the NPS Data

Source: Tanzania National Panel Survey (2012/13) and author's calculations

Analysing the prevalence of obesity by age groups, it is observed that prevalence is high among the middle-aged adults (35–64 years). This could probably be explained by the low level of metabolism for these age groups. However, the effect of SSB tax on obesity prevalence does not suggest a systematic pattern by age groups (Figure 5). The reduction of obesity is greater among those in age groups 25–34 years and 55–64 years, while there is no change in obesity prevalence among those above 65 years. This is probably because the young age group and the old age group have less income or tend to be more cautious with income allocation thus more likely to switching consumption after the tax introduction. Further exploration of the impact of SSB tax on BMI classes shows that the tax will reduce the prevalence of obesity and overweight (Figure 6). A reduction in SSB consumption is unlikely to increase amount of underweight people since the source of the calorie affects the quality of the nutrition (though not captured in the model). It is assumed that those who already have low caloric intake will adjust food intake if spending on SSBs reduces to have sufficient caloric intake.

### Sensitivity Analysis

Sensitivity analysis is undertaken to assess the effects of various tax rates and pass through rates on obesity prevalence. The results in Table 7 show, for different pass through rates, that the higher the SSB tax rate, the greater the reduction in the obesity prevalence for both males and females. There are therefore higher gains in terms of reduction of obesity prevalence with higher tax rates. However, this may also be associated with higher costs to producers and consumers as well.

**TABLE 7:** Sensitivity Analysis of the Effect of Changing Pass through Rate and SSB tax Rate on Obesity

Change in obesity prevalence (Percentage change)					
	Pass through rate	80	90	100	110
**Males**					
Tax rate	10	−6.5%	−7.3%	−7.3%	−7.3%
	20	−11.3%	−12.1%	−12.9%	−13.7%
	30	−16.1%	−18.5%	−20.2%	−21.8%
**Females**					
Tax rate	10	−2.0%	−2.2%	−2.3%	−2.3%
	20	−3.8%	−4.7%	−5.2%	−5.7%
	30	−5.9%	−7.0%	−8.2%	−8.6%

Source: Tanzania National Panel Survey (2012/13) and authors' calculations

### The Cost of Implementing the SSB Tax Intervention

As well as knowing how effective a tax is as a public health intervention, its cost-effectiveness should also be understood.^30^ The introduction or increase of a tax may bring in revenue from the tax, but implementation comes with its own administration cost. The due process for implementation of tax change in Tanzania should necessarily start with the finance ministry's fiscal (tax) policy decision regarding such matters as the relevant tax base or taxable item and the applicable tax rate. The policy choices made should then be enacted into law by parliament before the tax administration authority assumes responsibility for giving effect to the resulting legal provisions. This is the context in which the notion of implementation cost come. The approach by Lal and coauthors^18^ which considered the cost of passing legislation in parliament; administration and compliance time costs; field audit time costs; field audit direct costs; accountant yearly salary (government); and accountant yearly salary (industry) is adopted with some modification based on the practical realities. (The details of the estimation are provided as supplementary materials).

A total of TZS 69.1 million is estimated to be incurred in the first year of introduction of the fiscal (tax) policy intervention, comprising TZS 29.8 million as one-off cost in terms of preparation of the reform proposal and its passing into law, in the year of introduction of the reform. The other component comes in terms of continuous monitoring associated with increase in non-compliance risk arising from the additional/increased tax, estimated at TZS 39.3 million annually.

To permit a cost-benefit analysis, an estimate of tax revenue that will arise from the introduction of 20% increase in the tax rate on SSBs is also made. In computation of this estimate, it is assumed that there are two goods, SSBs and Substitutes for SSBs and that specific taxes () are imposed on the two goods (consistent with Tanzania's imposition practice in the area of beverages). The total revenue (R) from these excises can be obtained by:



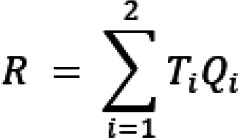



Assuming that the supply of the two goods is perfectly elastic, the amount of tax increase per unit is equal to the increase in demand price. That is,. If the tax levied on SSBs is increased, the change in the total tax revenue can be obtained by differentiating R with respect to T_i_



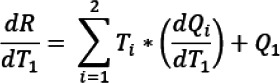



The percentage increase in total tax revenue can be calculated as



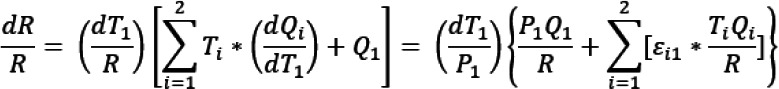



where stands for the demand elasticity of the ith goods with respect to the price of good 1 (SSBs).

From this formula, the total tax revenue is calculated and found to rise by 108.7% from TZS 416 Billion to TZS 868 Billion, an increase of TZS 452 Billion. Comparison of the TZS 452 billion increase in tax revenue to the administration cost in the year of introduction of the cost measure (TZS 69.1 million) results into a cost of collection ratio (as a measure of tax administration efficiency) of 0.02% (determined as cost of collection/tax revenue collected).

## DISCUSSION

Imposing a 20% SSB tax in Tanzania is predicted to reduce obesity by 6.6% overall and by 12.9% and 5.2% in adult males and adult females, respectively. The average overall reduction in energy intake is estimated to be 76.1 kJ per person per day. The SSB tax has more effect on adults at age group 15 to 34 and 55 to 64 years while it has no impact for those aged 65 years and above. The results obtained are similar to other studies such as Manyema and co-authors and Briggs and co-authors.^16,21^ It is projected that in 2020 the population of adult Tanzania's aged 15 years and above was around 32.65 million, of which 15.66 million are males and 16.99 are females. Using the baseline levels of obesity prevalence, this implies that there are about 423,000 males and 1.6 million females who are obese. The introduction of a 20% SSB tax will potentially reduce the number of obese people by about 47,000 among adult males and about 85,000 among adult females.

The SSBs tax already exists and that the proposed reform is essentially that of increasing the tax rates on an existing tax rather than introducing a new tax. Thus, the cost of administration to implement the proposed tax policy intervention is insignificant and the SSB tax can also potentially generate significant revenue.

### Strengths and Limitation to the Study

This is the first study in East Africa to model and quantify the potential effect of SSB tax on obesity. A number of studies have been conducted in developed countries^18,31,32^ and South Africa^16^ where the levels of obesity prevalence and NCDs are high but none have been done in East Africa where increasing levels of obesity and NCDs have been observed too. Despite several challenges with baseline consumption data of different drinks, this study has a number of strengths. Firstly, it uses nationally representative data for Tanzania to estimate the level of obesity prevalence in Tanzania which increases its external validity and generalisation. The BMI estimates were obtained through height and weight measured from the survey, which increased accuracy. Secondly, the model disaggregates the analysis by sex and age thus illustrates the differential effects of the proposed SSB tax by sex and age. Thirdly, this study has used Tanzania-specific own- and cross-elasticity data. The data enabled the disaggregation of the price elasticity by sex and age groups. Fourthly, the model used in this study took into account the substitution effect of the SSBs substitutes through the use of cross-price elasticities. This ensures that the reduction in total liquid caloric consumption is not overestimated. However, it was assumed that the substitution between the SSBs and foods is insignificant as suggested by the study by Finkelstein and co-authors.^17^

On the other hand, the study has some limitations. The first limitation of this study is that there was no available nationally representative data for consumption of SSBs and SSB substitutes. Second, the consumption and price estimates of SSBs were self-reported and they may have been affected by recall bias. Third, the data on SSBs' consumption could not capture all the SSBs consumed in Tanzania, rather the ones that are mostly consumed. Also, the data on SSBs' consumption could not specifically ascertain the amount of sugar content in each type of drink reported, therefore the study used the sugar content of the mostly consumed variety of drinks for each type of drink. Fourth, the study assumed a “full” pass through rate of the tax increase, but this may not be the case. Pass through rate may be different across age or income groups. Fifth, the model predicts a onetime tax effect of the changes in consumption of SSBs on body mass change. However, with persistent tax, the level of consumption will remain low and may also trigger behavior change which implies that the impacts may be underestimated. Sixth, this study has focused on the effect of SSB tax on obesity, while other NCDs' such as CVDs, diabetes, cancer have not been considered. Lastly, the study has not considered the effect of SSBs tax on non-health outcomes, such as disposable income and employment; and like other indirect taxes, this tax is likely to be regressive.

### Policy Implications

Our findings suggest that SSB tax is one of the strategies that can contribute to reversing the excess weight in the population and reduce obesity prevalence. The imposition of SSB tax should not, per se, be seen as a solution. It should be part of a broader approach complementing other strategies to reduce obesity prevalence and related NCDs such as promotion of physical activity and increased health promotion activities. Special attention should be given to women who already have a higher rate of obesity prevalence but are less affected by the tax compared to men to reduce their consumption of SSBs. Complex gender specific socioeconomic and cultural factors that increases women's risk of obesity need to be taken into account.

Secondly the study recommends that revenue raised from SSB tax should be dedicated to public health promotion programs including incentivizing production, supply and consumption of healthy foods such as fruits and vegetables, nutrition programs, improving the infrastructure that support increased physical activity and early detection of NCDs. Health care coverage especially, one related to NCDS, should be expanded at all levels of care starting from community health care programs and to a large extent be targeted to reach the poor who will be disproportionately affected by the SSB tax increase.

## CONCLUSION

There is limited specific recognition of sugar and SSBs as a huge contributor to NCDs despite the increasing evidence showing consumption of SSBs as a risk factor for obesity and diet related NCDs. (for example^11,13,14^) Diet related NCDs have become an increasing problem in our developing countries. Introduction of SSB taxation in Tanzania is a complex process that required evidence on the potential impact on obesity.

Recently, SSB taxes have been introduced in both developed and developing countries, such as France (2012), Mexico (2014), Berkeley, USA (2015), Mexico (2017) the United Kingdom (2018), Ireland (2018) and South Africa (2018). It has been documented that these taxes have led to the drop in the households purchase of sugary drinks for the general population especially for for the poor.^33,34^ It is also documented that the reduction was substituted by an increase in sales of light/zero drinks and that the reduction in purchases was stronger in areas with a higher incidence of obesity, higher household incomes and for products with higher sugar content. ^34^

The experience in South Africa shows that habitual and addictive behavior towards consumption of SSBs, fueled by mass advertising campaigns and wide accessibility of SSB requires the introduction of SSB tax to be complemented with a multipronged behaviour change strategy.^35^

The findings of this study show that an SSB tax in Tanzania will lead to reduction in the average overall energy intake and consequently an overall reduction in prevalence of obesity. It is practically feasible to introduce SSB tax beyond the existing excise tax in Tanzania, since the system is already existing and what remains is to increase the rate. The challenge will be on the stakeholders and public support and understanding of the aim of the proposed tax increase. Although there is a general recognition of NCDs as an emerging problem across the board, there is still an imbalance between public health concerns and commercial and economic interests. The soft drink industry is economically powerful and has strong lobbying power. The very influential industry may diminish the feasibility of introducing SSB tax.

Further, its implementation requires active involvement of all the stakeholders guided by evidence-based policies of implementation, monitoring and evaluation and this should be done within the parameter of the country's legal framework. The importance of policy champions in Tanzania policy making context cannot be understated in boosting political commitment on NCDs. There has been lack of active civil society engagement in the fight against SSBs though they have a big opportunities and role to strengthen this effort.

## References

[1] World Health Organization. Noncommunicable Diseases Country Profiles. World Health Organization; 2018.

[2] World Health Organization. Global Status Report on Noncommunicable Diseases 2014 (No. WHO/NMH/NVI/15.1). World Health Organization; 2014.

[3] Abubakari AR, Lauder W, Agyemang C, Jones M, Kirk A, Bhopal RS. Prevalence and time trends in obesity among adult West African populations: a meta-analysis. Obesity Reviews. 2008;9(4):297–311. doi:10.1111/j.1467-789X.2007.00462.x 18179616

[4] Kimani-Murage EW, Kahn K, Pettifor JM, Tollman SM, Klipstein-Grobusch K, Norris SA. Predictors of adolescent weight status and central obesity in rural South Africa. Public Health Nutr. 2011;14(6):1114–1122. doi:10.1017/S1368980011000139 21356151 PMC3370923

[5] World Bank. World Development Indicators 2016. World Bank; 2016.

[6] United Republic of Tanzania. STEPS Survey of NCD Risk Factors; 2012.

[7] Mayige M, Kagaruki G, Ramaiya K, Swai A. Non communicable diseases in Tanzania: a call for urgent action. Tanzan J Health Res. 2012;13(5). doi:10.4314/thrb.v13i5.7 26591992

[8] Mfinanga SG, Kivuyo SL, Ezekiel L, Ngadaya E, Mghamba J, Ramaiya K. Public health concern alongside with global initiative on the priority action for “silent uprising epidemic” on Non-Communicable Diseases in Tanzania. Tanzania Journal of Health Research March 2012;13(5): 6. DOI:10.4314/thrb.v13i5.6 26591991

[9] Kankeu HT, Saksena P, Xu K, Evans DB. The financial burden from non-communicable diseases in low- and middle-income countries: a literature review. Health Res Policy Syst. 2013;11(1):31. doi:10.1186/1478-4505-11-31 23947294 PMC3751656

[10] Murray CJ, Aravkin AY, Zheng P, et al. Global burden of 87 risk factors in 204 countries and territories, 1990–2019: a systematic analysis for the Global Burden of Disease Study 2019. Lancet. 2020; 396(10258), pp. 1223–1249. doi: 10.1016/S0140-6736(20)30752-2 33069327 PMC7566194

[11] Vartanian LR, Schwartz MB, Brownell KD. Effects of Soft Drink Consumption on Nutrition and Health: A Systematic Review and Meta-Analysis. Am J Public Health. 2007;97(4):667–675. doi:10.2105/AJPH.2005.083782 17329656 PMC1829363

[12] Chen L, Appel LJ, Loria C, et al. Reduction in consumption of sugar-sweetened beverages is associated with weight loss: the PREMIER trial. Am J Clin Nutr. 2009;89(5):1299–1306. doi:10.3945/ajcn.2008.27240 19339405 PMC2676995

[13] Malik VS, Schulze MB, Hu FB. Intake of sugar-sweetened beverages and weight gain: a systematic review. Am J Clin Nutr. 2006;84(2):274–288. doi:10.1093/ajcn/84.2.274 16895873 PMC3210834

[14] Hu FB, Malik VS. Sugar-sweetened beverages and risk of obesity and type 2 diabetes: Epidemiologic evidence. Physiol Behav. 2010;100(1):47–54. doi:10.1016/j.physbeh.2010.01.036 20138901 PMC2862460

[15] Popkin BM, Duffey KJ. Sugar and Artificial Sweeteners: Seeking the Sweet Truth. In: Nutrition Guide for Physicians. Humana Press; 2010: 25–38. doi:10.1007/978-1-60327-431-9_3

[16] Manyema M, Veerman LJ, Chola L, et al. The Potential Impact of a 20% Tax on Sugar-Sweetened Beverages on Obesity in South African Adults: A Mathematical Model. PLoS One. 2014;9(8):e105287. doi:10.1371/journal.pone.0105287 25136987 PMC4138175

[17] Finkelstein EA, Zhen C, Bilger M, Nonnemaker J, Farooqui AM, Todd JE. Implications of a sugar-sweetened beverage (SSB) tax when substitutions to non-beverage items are considered. J Health Econ. 2013;32(1):219–239. doi:10.1016/j.jhealeco.2012.10.005 23202266

[18] Lal A, Mantilla-Herrera AM, Veerman L, et al. Modelled health benefits of a sugar-sweetened beverage tax across different socioeconomic groups in Australia: A cost-effectiveness and equity analysis. PLoS Med. 2017;14(6):e1002326. doi:10.1371/journal.pmed.1002326 28654688 PMC5486958

[19] Claro RM, Levy RB, Popkin BM, Monteiro CA. Sugar-Sweetened Beverage Taxes in Brazil. Am J Public Health. 2012;102(1):178–183. doi:10.2105/AJPH.2011.300313 22095333 PMC3490548

[20] Briggs AD, Mytton OT, Madden D, O'Shea D, Rayner M, Scarborough P. The potential impact on obesity of a 10% tax on sugar-sweetened beverages in Ireland, an effect assessment modelling study. BMC Public Health. 2013;13(1):860. doi:10.1186/1471-2458-13-860 24044370 PMC3852031

[21] Briggs ADM, Mytton OT, Kehlbacher A, Tiffin R, Rayner M, Scarborough P. Overall and income specific effect on prevalence of overweight and obesity of 20% sugar sweetened drink tax in UK: econometric and comparative risk assessment modelling study. BMJ. 2013;347(oct 31 4):f6189–f6189. doi:10.1136/bmj.f6189 24179043 PMC3814405

[22] Cabrera Escobar MA, Veerman JL, Tollman SM, Bertram MY, Hofman KJ. Evidence that a tax on sugar sweetened beverages reduces the obesity rate: a meta-analysis. BMC Public Health. 2013;13(1):1072. doi:10.1186/1471-2458-13-1072 24225016 PMC3840583

[23] Basu S, Vellakkal S, Agrawal S, Stuckler D, Popkin B, Ebrahim S. Averting Obesity and Type 2 Diabetes in India through Sugar-Sweetened Beverage Taxation: An Economic-Epidemiologic Modeling Study. PLoS Med. 2014;11(1):e1001582. doi:10.1371/journal.pmed.1001582 24409102 PMC3883641

[24] Besley TJ, Rosen HS. Sales Taxes and Prices: An Empirical Analysis. Natl Tax J. 1999;52(2):157–178. doi:10.1086/NTJ41789387

[25] Berardi N, Sevestre P, Tépaut M, Vigneron A. The impact of a ‘soda tax’ on prices: evidence from French micro data. Appl Econ. 2016;48(41):3976–3994. doi:10.1080/00036846.2016.1150946

[26] Bahl R, Bird R, Walker MB. The Uneasy Case Against Discriminatory Excise Taxation: Soft Drink Taxes in Ireland. Public Finance Review. 2003;31(5):510–533. doi:10.1177/1091142103253753

[27] Crawford I, Keen M, Smith S. Value added tax and excises. Dimensions of tax design. In: Mirrlees JA, Adam S, eds. Dimensions of Tax Design: The Mirrlees Review. Oxford University Press; 2010: 275–362.

[28] Swinburn BA, Sacks G, Lo SK, et al. Estimating the changes in energy flux that characterize the rise in obesity prevalence. Am J Clin Nutr. 2009;89(6):1723–1728. doi:10.3945/ajcn.2008.27061 19369382 PMC3738432

[29] Hall KD, Sacks G, Chandramohan D, et al. Quantification of the effect of energy imbalance on bodyweight. The Lancet. 2011;378(9793):826–837. doi:10.1016/S0140-6736(11)60812-X PMC3880593 21872751

[30] Wilson N, Nghiem N, Foster R, Cobiac L, Blakely T. Estimating the cost of new public health legislation. Bull World Health Organ. 2012;90(7):532–539. doi:10.2471/BLT.11.097584 22807599 PMC3397705

[31] Andreyeva T, Chaloupka FJ, Brownell KD. Estimating the potential of taxes on sugar-sweetened beverages to reduce consumption and generate revenue. Prev Med (Baltim). 2011;52(6):413–416. doi:10.1016/j.ypmed.2011.03.013 21443899

[32] Dharmasena S, Capps O. Intended and unintended consequences of a proposed national tax on sugar-sweetened beverages to combat the U.S. obesity problem. Health Econ. 2012;21(6):669–694. doi:10.1002/hec.1738 21538676

[33] Colchero MA, Rivera-Dommarco J, Popkin BM, Ng SW. In Mexico, Evidence Of Sustained Consumer Response Two Years After Implementing A Sugar-Sweetened Beverage Tax. Health Aff. 2017;36(3):564–571. doi:10.1377/hlthaff.2016.1231 PMC5442881 28228484

[34] Vall Castelló J, Lopez Casasnovas G. Impact of SSB taxes on sales. Econ Hum Biol. 2020;36:100821. doi:10.1016/j.ehb.2019.100821 31654894

[35] Bosire EN, Stacey N, Mukoma G, Tugendhaft A, Hofman K, Norris SA. Attitudes and perceptions among urban South Africans towards sugar-sweetened beverages and taxation. Public Health Nutr. 2020;23(2):374–383. doi:10.1017/S1368980019001356 31179956 PMC10200411

